# COVID-19 Disease Leading to Chronic Spontaneous Urticaria Exacerbation: A Romanian Retrospective Study

**DOI:** 10.3390/healthcare9091144

**Published:** 2021-09-01

**Authors:** Ioana Adriana Muntean, Irena Pintea, Ioana Corina Bocsan, Carmen Teodora Dobrican, Diana Deleanu

**Affiliations:** 1 Department of Allergology and Immunology, “Octavian Fodor” Institute of Gastroenterology and Hepatology, “Iuliu Hatieganu” University of Medicine and Pharmacy, 400462 Cluj Napoca, Romania; Adriana.Muntean@umfcluj.ro (I.A.M.); diana.deleanu@umfcluj.ro (D.D.); 2 Department of Pharmacology, Toxicology and Clinical Pharmacology, “Iuliu Hatieganu” University of Medicine and Pharmacy, 400462 Cluj Napoca, Romania; bocsan.corina@umfcluj.ro

**Keywords:** COVID-19, chronic spontaneous urticaria, SARS-CoV-2 infection, mast cells

## Abstract

(1) Background: The COVID-19 pandemic has resulted in the exacerbation of various chronic diseases. Due to the potential impact of SARS-CoV-2 on mast cells, we aimed to analyze the relevance of COVID-19 disease on chronic spontaneous urticaria (CSU) clinical presentation and biological profile. (2) Methods: This study is a retrospective case series of patients with CSU diagnosed and treated in the Allergy Department of the Professor Doctor Octavian Fodor RIGH, (Cluj-Napoca, Romania). Patients were assessed for disease activity and level of control with the weekly urticaria activity score and the visual analogue scale. Results were correlated with COVID-19 severity and with nonspecific markers of inflammation during and after the SARS-CoV-2 infection. (3) Results: SARS-CoV-2 impacted a significant proportion (33%) of the CSU patients, of which 71% developed a moderate-severe form of COVID-19. Most of the patients (68%) had moderate-severe forms of CSU and 65% took AH1 treatment (one dose, two-fold dose or four-fold dose). The rest of them (35%) received the second-line treatment (40.3% Omalizumab, 53% Prednisolone and 4.8% Cyclosporine). In Omalizumab treated group of UCS patients we observed that COVID-19 disease was not severe. We established a positive correlation between the severity of the infection and that of the CSU clinical presentation, with most bothersome symptoms of urticaria being experienced by moderate to severe COVID-19 CSU patients (47%). Inflammatory markers were positively correlated (*p* = 0.01) with a more severe clinical profile of CSU, in accordance with our hypothesis that the level of inflammation triggered by COVID-19 disease has a role in CSU exacerbation. The non-specific inflammatory markers, such as CRP, were positively associated with the UAS7 score (R2 = 0.363; *p* = 0.001). An increased rate of exacerbation of CSU was observed in moderate-severe COVID-19 infection. (4) Conclusions: COVID-19 disease can result in the exacerbation of chronic spontaneous urticaria, more likely in moderate to severe forms of infection.

## 1. Introduction

Chronic spontaneous urticaria (CSU) is a disease characterized by the recurrence of pruritic wheals, occurring on most days of the week, for longer than six weeks, and accompanied by angioedema in more than 50% of the cases. In some patients, angioedema is the only clinical feature of the disease [1]. Although CSU is a limited disorder in most cases, with an average duration of disease of two to five years, active and/or refractory, difficult to treat cases pose a difficult challenge to patients and clinicians alike [1,2]. Active chronic spontaneous urticaria is a debilitating disorder, which has a major impact on the quality of life of affected individuals and is a substantial global burden [3].

The current CSU treatment algorithm follows a stepwise approach, starting with standard doses of second-generation non-sedative H1 antihistamines as the first line treatment. Up-dosing of up to a 4-fold increase of H1 antihistamines in cases non-responsive after 2–4 weeks of first line treatment, or earlier. In antihistamine-resistant patients, treatment with Omalizumab and, if this fails, Cyclosporine is the current guideline-recommended therapy of choice [1,2,3].

There are several theories regarding the pathogenesis of CSU, mainly involving autoinflammation and mast cell (MCs) mediator release. MC degranulation is a central event in the development of CSU cutaneous lesions, and histamine levels are elevated in biopsy skin samples [3]. MCs are strategically placed at sites that interface with our external environment such as the skin, lung, and intestines. These locations allow them to act as sentinels for tissue damage and pathogen invasion. The association between MCs and blood vessels is optimal to enhance the rapid recruitment of effector cells out of the bloodstream and into neighboring tissues [4].

Since the beginning of 2020, severe acute respiratory coronavirus 2 (SARS-CoV-2) has spread rapidly across the globe, causing the “coronavirus disease 2019” (COVID-19) pandemic, the worst global public health crisis. The pathology of severe COVID-19 is characterized by elevated levels of proinflammatory cytokines, mainly tumor necrosis factor-alpha (TNF-α), interleukin-6 (IL-6), IL-1β, granulocyte-macrophage colony-stimulating factor (GM-CSF), and chemokine (C-C-motif) ligand 2 (CCL2), many of which are produced and released by MCs [4,5,6]. There is evidence for SARS-CoV-2 and MCs’ activation. MCs, key effector cells in CSU, along with other immune cells such as basophils, neutrophils, monocytes/macrophages, and natural killer cells are involved in cytokine storms triggered by SARS-CoV-2 severe infection [7,8]. In addition, MCs can recognize and respond to viruses through several different receptors, including toll-like receptors, retinoic acid–inducible gene-I–like receptors, FcεRI, complement, and IL-1 receptors. Engagement of these receptors results in MCs’ activation and degranulation. This process is facilitated by the MCs’ rapid production of proinflammatory cytokine mediators such as TNF and IL-1β that activate endothelium, leukotrienes and prostaglandins that facilitate vasodilatation, as well as a range of chemokines that promote selective recruitment of specific subsets of effector cells [5]. Moreover, recent studies show that human mast cells can be synergistically stimulated by the peptide substance P and IL-33 to release impressive amounts of vascular endothelial growth factor, IL-1β or tumor necrosis factor again without secretion of histamine or tryptase. In addition, mast cells express the renin-angiotensin system, the ectoprotease angiotensin-converting enzyme 2 required for SARS-CoV-2 binding, and serine proteases, including TMPRSS2, required for priming of the corona spike protein [9].

Recent studies bring arguments in favor of CSU exacerbation in the context of COVID-19 [10]. Due to the potential impact of the acute respiratory syndrome coronavirus 2 (SARS-CoV-2) on inflammation in general and on mast cells, the key effector cells in chronic spontaneous urticaria (CSU) we analyzed the relevance of SARS-CoV-2 infection on CSU clinical presentation and biological profile.

## 2. Materials and Methods

### 2.1. Study Design

This study is a retrospective case series of patients (*n* = 218) with CSU diagnosed and treated in the Allergy Department of the Professor Doctor Octavian Fodor Regional Institute of Gastroenterology and Hepatology, (Cluj-Napoca, Romania), between February 2017 and April 2021, since January 2020 this group of patients were evaluated for the presence of COVID-19 disease and UCS exacerbations. The study protocol was approved by the “Octavian Fodor” Institute of Gastroenterology and Hepatology Ethics Committee, and all patients signed the informed consent before any assessments were done. Inclusion criteria were the presence of CSU recent onset or personal history of CSU in the last 5 years. The exclusion criteria were as follows: known food allergies, inadequate control of chronic thyroid diseases (normal TSH level at initial evaluation), and concomitant chronic inflammatory disease: asthma, COPD, systemic autoimmune diseases, chronic infections (e.g., viral B hepatitis, viral C hepatitis which continue to show an increased prevalence in Romania) [11].

### 2.2. Patients and Clinical Evaluation

Diagnosis of CSU was done according to the EAACI/GA2LEN/EDF/WAO guideline. Diagnosis was based on history and typically symptoms: wheals and itch associated with or without angioedema. Age, sex, and region (rural/urban) and urticaria related symptoms were recorded. Patients were assessed for disease activity, impact, and level of control with the weekly urticaria activity score (UAS7), and the Visual Analog Scale (VAS). Interviews with the patients were made by phone or online and face to face during a clinical exam in severe symptomatic patients.

Urticaria Activity Score (UAS7) evaluated the number of wheals and itching. The number of wheals was counted by the patients and analyzed on a scale from 0 to 3 (0 = absent, 1 ≤ 20 wheals over 24 h, 2 = 20–50 wheals over 24 h, 3 ≥ 50 wheals over 24 h for), for 7 days. Itching was analyzed on a scale from 0 to 3 (0 = absent, 1 = mild, 2 = moderate, 3 = severe), retrospectively for 24 h, for 7 days. Patients with UAS7 values that were higher than 16 were included in the moderate-severe CSU group. Also, VAS scale was used to assess the QL (quality of life), and a VAS value over 7 points indicated patients with moderate/severe form of CSU. Visits were performed via e-mail or via telephone, and patients completed the VAS and UAS7 monthly, at home, and sent the results by text message or e-mail. Some of the patients required a face-to-face consultation following COVID-19 disease, due to CSU severity, to increase the step of CSU treatment.

### 2.3. COVID-19 Disease

COVID-19 disease severity was defined by using the scale provided by the World Health Organization, according to data from previous presentations at the Infectious Disease Hospital in Cluj-Napoca. Clinical direct evaluation was performed after the 14 days of quarantine required for SARS-CoV-2 infection. All the patients were called and advised to inform in case they contracted the virus.

In all CSU patients who contracted the virus, the first line of treatment for CSU consisted of AH1 up to 4-fold dose and a prolonged course of prednisolone of 2–4 weeks, which was sufficient to control the symptoms of CSU, with only one non-responder, who was introduced on Omalizumab 300 mg monthly after the 4 weeks of conventional treatment failed to improve/control the disease. This patient responded well to the anti-IgE therapy and remission of CSU was obtained 4 months after the initiation of the biological therapy.

### 2.4. Biological Evaluation

Blood tests performed in all patients were: complete blood count, CRP, LDH, Troponin, TSH. These tests were done in a laboratory with national accreditation. A jeun blood samples were collected. The samples were collected on days 10 to 15 after the positive SARS-CoV-2 rtPCR.

### 2.5. Statistical Analysis

The statistical analysis was performed using Microsoft Excel and SPSS version 21 (Chicago, IL, USA). Data were labeled as nominal, expressed as continuous variables. Variables with normal distribution were expressed as mean and standard deviation. The differences were placed in groups using the Wilcoxon Signed Rank test and between groups using the Mann Whitney test. The Spearman’ coefficient of correlation was calculated to highlight differences between continuous variables. The level of statistical significance was set at *p* < 0.05.

## 3. Results

### 3.1. Patients’ Demographic Data

In Table 1 we analyzed demographic data of our patient’s group. Median age was 48.3 (23–75) years and the sex ratio M:F was 1:3. 68% had moderate-severe forms of CSU and in this group the medium age was significantly higher (*p* = 0.01). Patients with a UAS7 score greater than 16 were included in moderate-severe forms of CSU. UAS7 and VAS were positively associated and statistically significant (R = 0.138, *p* = 0.01). Also, treatment with Omalizumab, Cyclosporine and Prednisone is found only in moderate/severe CSU patients.

### 3.2. The Treatment in Our Group with CSU

**Figure 1:**
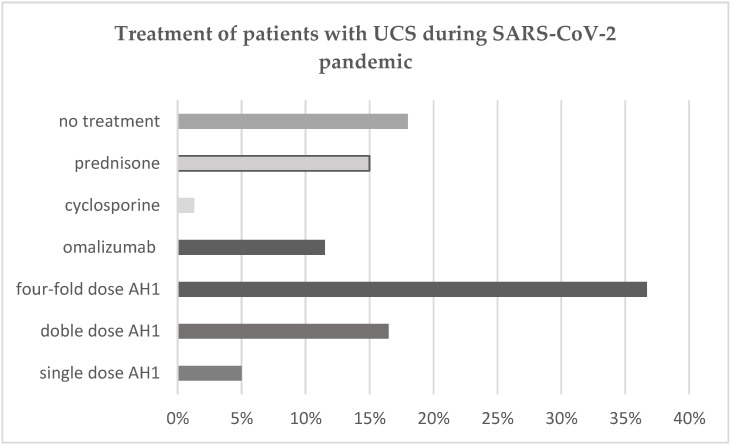
Treatment in patients with CSU during SARS-CoV-2 pandemic, represented as percent.

The CSU treatment options for patients during the SARS-CoV-2 pandemic (February 2020–April 2021) under our care are presented in Figure 1. Most of the patients (68%) had moderate-severe forms of CSU and 65% took AH1 treatment (one dose, two-fold dose or four-fold dose). The rest of them (35%) received the second-line treatment. Treatment with omalizumab and cyclosporine is not supported by the national healthcare system in Romania. Due to high costs, some of the patients who suffer from refractory CSU, and are thus non-responsive to AH1, cannot afford to pay for the therapy. These patients received a prednisone course. Patients with uncontrolled CSU during SARS-CoV-2 pandemic received treatment according to the CSU guideline (40.3% Omalizumab, 53% Prednisolone, and 4.8% Cyclosporine). Prednisone was the most frequent drug used in managing CSU flares during SARS-CoV-2 pandemic. The dose of Omalizumab was 300 mg/month followed by a 6 month course of treatment. Cyclosporine was given with a dose of 3 mg/body weight for 2 months, 2 mg/body weight for 1 month, and 1 mg/body weight for 3 months [10], while the Prednisone course started with 0.5 mg/kg for 1 week, then the dose was tapered with 5 mg/day until a dose of 10 mg/day was reached and maintained for one week, followed by 10 mg every 2 days for a month or more in nonresponsive patients who couldn’t afford/refused the omalizumab or cyclosporine therapy.

### 3.3. CSU and COVID-19 Disease

Only 33% of patients from the CSU group went through COVID-19, of which 71% developed a moderate-severe form of disease. During SARS-CoV-2 infection, the patients were in different states of treatment for CSU: no treatment, single dose of AH1, double dose of AH1, four-fold dose of AH1 and omalizumab, cyclosporine and prednisone as add-on treatment to four-fold AH1, as described in Table 2.

During and after COVID-19 disease, the course of CSU symptoms modified, such that: 44% of infected patients had an increased severity of the disease, more frequently seen in moderate-severe COVID infection—47%), but CSU did not influence the severity of COVID infection, as described in Table 3. Most likely the high degree of inflammation in moderate-severe COVID-19 disease plays an important role in CSU exacerbation.

The non-specific inflammatory markers, such as CRP, were positively associated with the UAS7 score (R2 = 0.363; *p* = 0.001), and all the markers were significantly increased in moderate-severe COVID-19 infection group of patients, as described in Table 4, with an increased rate of exacerbation of CSU in moderate-severe COVID-19 infection.

In the Omalizumab group, which received 4-fold dose AH1 daily and 300 mg Omalizumab monthly; the rate of CSU exacerbation was low: 1 case from the 10 infected (1:10).

In the AH1-only treatment and no treatment groups the rate of CSU exacerbation was 28 cases from the 62 infected (1:2).

## 4. Discussion

The severe acute respiratory syndrome coronavirus 2, known as coronavirus disease 2019 (COVID-19), is associated with high morbidity and mortality mostly in adults, owing to systemic symptoms as well as to the exacerbation of several chronic diseases, CSU included [4].

CSU is a chronic condition that often comes as a fluctuating disease with activity and remissions, negatively impacting the QL of patients. Since more than 50% of the patients have insufficient treatment response to antihistamines, CSU proves to be a debilitating disease and a challenge to affected individuals and clinicians alike [1,2].

The pathological findings associated with COVID-19 seem to result from the release of numerous proinflammatory molecular mediators. Mast cells, key effector immune cells in the secretion of such cytokines and chemokines, are ubiquitous in the body, especially the skin, and are critical for CSU pathology [12]. Both COVID-19 disease and CSU triggered an important inflammatory process in the whole organism.

Mast cells are typically activated by allergic triggers, but they can also be triggered by pathogen-associated molecular patterns via activation of toll-like receptors. In CSU, MCs mediators’ release has various triggers: acute infections, fever, NSAIDs, histamine containing food, physical factors, such as pressure, cold, heat, exercise or sun exposure, alcohol, premenstrual or ovulatory phase, to name a few. Thus, symptoms associated to mast cell degranulation may also appear in some viral infections [1]. Mast cells (MCs) play an important role in the immune response because when they recognize viral products, they are activated and synthesize many chemokines and cytokines [13]. In addition, some cytokines secreted by other cells such as T cells, damaged epithelial and endothelial cells or even by themselves stimulated MC activation as Hermans et al. suggested (2019) [14]. The role of MCs in coronavirus-induced disease have been discussed since the beginning of The Toll-like receptor 3 detection of viral double-stranded ribonucleic acid (RNA), viral sphingosine-1-phosphate (S1P) binding to S1P receptors, and retinoic acid-induced gene I (RIG-I) recognition of uncapped viral RNA) [6], they also express angiotensin converting enzyme 2 (ACE2), now known as the principal receptor for SARS-CoV-2, thus defining a route by which MCs could also become hosts for this virus [9].

It is well known that most SARS-CoV-2 positive patients are asymptomatic or have mild symptoms. However, increasing evidence suggests that many patients who either recovered from or had mild symptoms after COVID-19 exhibit diffuse, multiorgan symptoms months after the infection, known as the adult multisystem inflammatory syndrome [15]. The clinical picture includes symptoms that vary within wide ranges, from malaise, myalgias, chest tightness, brain fog to neuropsychiatric symptoms that are similar to those of patients with mast cell activation syndrome [16].

The present study aimed to analyze the impact of COVID-19 disease on CSU patients in our care. Our study included a number of 218 patients with CSU found in our care, in the context of the SARS-CoV-2 pandemic. 18% of our CSU patients were in remission of the disease, and those were contacted only for COVID-19 disease evaluation. Majority of our patients were on four-fold AH1 dose (36%) which differs from other studies where sedating AH1 were used and in non-responsive patients Omalizumab was recommended, similarly to AWARE-study [17].

During the pandemic, the use of AH1 in our patients is high, and most of the patients used those drugs, due to drug availability, as well as reduced access to costly biological therapy. However, there was one patient in whom we successfully administered Omalizumab in CSU exacerbated by COVID-19, which rapidly improved the UAS7 and VAS scores. Recent studies bring arguments in favor of the clinical benefit of anti-IgE therapy in individuals with CSU exacerbated by COVID-19 [18,19,20]. Moreover, in our study, the most severe exacerbations of CSU were particularly seen in patients treated with AH1 alone and in those who had no treatment need for CSU prior to COVID-19 disease (in half of patients in this group CSU was exacerbated by COVID-19 disease). We hypothesize that this is due to an uncontrolled subclinical systemic inflammation in patients treated with AH1 alone, triggered by infection. Although current data prove that AH1 exhibits a mild systemic anti-inflammatory effect [21], this effect is too low in the case of viral infections triggering CSU when short courses of systemic corticosteroids are frequently used, as in our study. Also, systemic corticosteroids are used in moderate/severe COVID-19 disease [19], so that could be the reason for some patients feeling better (9.8%) and some having an unchanged course of CSU (43.1%). Although, analyzing the main treatment of COVID-19 infected patients was not our objective for this study, we may hypothesize that in COVID-19 disease treated with systemic or oral corticosteroids may also improve CSU due to the common anti-inflammatory mechanism, effective in both diseases.

All these data plead for the complex interplay between the SARS-CoV-2 and the mast cells of the host, and probably only severe disease will have an augmentation of the systemic inflammation leading to CSU exacerbation; in our study we observed that 70% of patients had a moderate to severe form of COVID-19 disease.

According to our findings, the pandemic severely impaired CSU patient care and evolution. Only 33% of the patients from the CSU group went through COVID-19 disease of which 71% developed a moderate-severe form of disease. During and after COVID-19 disease, a significant proportion of patients (44%) experienced a more severe clinical profile of CSU. The severity of CSU did not influence the course of COVID-19 disease as other studies showed [10], despite the treatment of CSU. During SARS-CoV-2 infection the patients were in different states of treatment for CSU: no treatment, single dose of AH1, double dose of AH1, four-fold dose of AH1 and omalizumab, cyclosporine and prednisone as add-on treatment to four-fold AH1, as described in Table 2. Since in our COVID-19 disease patients’ group there was none with cyclosporine treatment, which is an important immunosuppressor drug, we cannot make any comments on this situation. In the omalizumab group: this group received four-fold dose AH1 daily and 300 mg omalizumab monthly; the rate of CSU exacerbation was low: 1 case from the 10 infected (1:10). In the AH1-only treatment and no treatment groups the rate of CSU exacerbation was 28 cases from the 62 infected (1:2). We may hypothesize that maybe omalizumab has a better effect on inflammation and a protective role for exacerbation in CSU [22,23]. In our patients with CSU treated with omalizumab, the course of COVID-19 disease was not observed to be more severe, as described in recent study of Passante M. et al. [24].

Moreover, we established a positive correlation between the severity of the infection and that of the CSU clinical presentation, with most bothersome symptoms of urticaria being experienced by moderate to severe COVID-19 CSU patients (47%), which was proved by the UAS7 scores and VAS values. Although VAS is a tool used for assessing the QoL in Allergic Rhinitis corelated with RQLQ (Rhinitis Quality of Life Questionnaire), it is very simple to evaluate the impact of a chronic diseases [25] and it could also be an efficient tool to assess CSU impact on QoL. In our study UAS7 and VAS were positively and statistically significant correlated (R = 0.138, *p* = 0.01). But to validate VAS as a tool in CSU a multicenter study with higher number of patients is necessary.

In addition, inflammatory markers were positively correlated with the UAS7 score in moderate to severe SARS-CoV-2 infected group of CSU patients, in compliance with our hypothesis that the level of inflammation triggered by COVID-19 disease has a role in CSU exacerbation. Inversely, the severity of CSU did not influence that of COVID infection. No COVID-19 death was reported in our group of CSU patients. This is consistent with recent studies showing preponderance for CSU exacerbation in the context of COVID-19 disease [10,26].

Our study shows that systemic inflammation evaluated by nonspecific markers is statistically significantly higher in moderate to severe COVID-19 than in mild forms of disease. The markers we used were CRP, troponin, LDH, which are involved in urticaria exacerbation, as shown by other studies [27]. CRP was positively associated with the UAS7 score (R2 = 0.363; *p* = 0.001), so the more inflammation there is, the more impairment of CSU symptoms patients experience. In addition, some data plead for the value of CRP as a biomarker that correlates with the activity of CSU, which is consistent with our result.

Limitations of our study consist in the fact that patients with mild COVID-19 infection had inflammatory markers evaluated only after 14 days of quarantine, while in severe patients that had multiple blood samples determinations from which we took a medium value in this analysis.

The value of the study results from analyzing the impact of COVID-19 disease in a small group of CSU patients from a region not included in European Analysis. It is the first study showing the possibility to use VAS as a QoL measurement in CSU.

## 5. Conclusions

Mast cells, key effector immune cells in chronic spontaneous urticaria, could also contribute to the pathogenesis of COVID-19 and any post infectious inflammatory syndromes through the release of proinflammatory mediators. Thus, COVID-19 disease can result in the exacerbation of chronic spontaneous urticaria, more likely in moderate to severe forms of infection, which may lead to high health and financial costs associated with CSU. Given these data, we plead in favor of blocking mast cells and the action of their mediators both prophylactically and symptomatically during the COVID-19 pandemic in susceptible patients, CSU included.

## Figures and Tables

**Table 1:** Demographic characteristics of the patients in our study group.

Parameter		Mild CSU (*n* = 69)	Moderate/Severe CSU (*n* = 149)	*p*
Age *		38.05 ± 6.2	59.89 ± 9.7	0.01
Sex	male	34.7% (24)	20.1% (30)	0.2
	female	65.3% (65)	79.9% (99)	
Living area	urban	78.2% (54)	68.4% (102)	0.7
	rural	21.8% (15)	31.6% (27)	
CSU onset (months)		124 (3–160)	186 (4.5–268)	0.5
CSU scores	UAS7	≤15	≥16	0.01
	VAS	≤6	≥6	0.01
Treatment	AH1	10% (7)	2.6% (4)	0.05
	2xAH1	37.6% (26)	5.9% (10)	0.8
	4xAH1	37.6% (26)	36.2% (54)	0.05
	* Omalizumab	0% (0)	16.7% (25)	0.01
	* Cyclosporine	0% (0)	2% (3)	0.01
	* Prednisone	0% (0)	22.1% (33)	0.01
CSU in remission	No treatment	21.8% (15)	16.7% (25)	0.6
COVID 19 infection	Not present	69.5% (48)	65.7% (98)	0.7
	Present	31.5% (21)	34.3% (51)	0.8

* Data are expressed as mean ± SD. Significance *p* < 0.05.

**Table 2:** CSU treatment during COVID-19 mild or moderate-severe type of disease, represented as percent and number of patients.

CSU Treatment	Mild COVID-19 Infection (*n* = 21)	Moderate/Severe COVID-19 Infection (*n* = 51)
Single dose AH1	14.2% (3)	1.9% (1)
Double dose AH1	42.8% (9)	29.4% (15)
Four-fold dose AH1	33.3% (7)	39.1% (20)
Omalizumab	0% (0)	19.6% (10)
Cyclosporine	0% (0)	0% (0)
Prednisone	0% (0)	3.9% (2)
No treatment	9.7% (2)	9.1% (5)

**Table 3:** CSU course during COVID-19 mild or moderate-severe infection represented as percent and number of patients.

CSU Course	Mild COVID-19 Infection (*n* = 21)	Moderate/Severe COVID-19 Infection (*n* = 51)
Better	4.7% (1)	9.8% (5)
No change	61.9% (13)	43.1% (22)
Worse	33.4% (7)	47% (24)

**Table 4:** Inflammation markers in CSU patient during COVID-19 mild or moderate-severe infection.

Parameter	Mild COVID-19 Infection	Moderate/Severe COVID-19 Infection	*p*
Leucocytes * 10^3^/μL	7.54 (±9.7)	3.89 (±2.9)	0.03
CRP * (mg/dL)	2.9 (±6.1)	49.8 (±52.5)	0.01
Troponin * (μg/L)	0.1 (±0.2)	2.3 (±1.8)	0.02
LDH * (U/L)	438 (±195.6)	657 (±185.8)	0.05
UAS7	23 (±5.2)	38 (±3.8)	0.03
VAS	7 (±1.4)	9 (±0.8)	0.02

* Data are expressed as mean ± SD.

## Data Availability

Data are available at Allergology Department, Octavian Fodor” Institute of Gastroenterology and Hepatology, Cluj Napoca.
